# Can achievement goal theory provide a useful motivational perspective for explaining psychosocial attributes of medical students?

**DOI:** 10.1186/1472-6920-12-4

**Published:** 2012-01-12

**Authors:** Nir Madjar, Yaacov G Bachner, Talma Kushnir

**Affiliations:** 1 Department of Education, Ben Gurion University of the Negev, Beer Sheva, Israel; 2 Department of Sociology of Health, Faculty of Health Sciences, Ben Gurion University of the Negev, Beer Sheva, Israel

## Abstract

**Background:**

Psychosocial competence and frustration tolerance are important characteristics of skilled medical professionals. In the present study we explored the usefulness of applying a comprehensive motivational theory (Goal orientations), for this purpose. According to goal orientation theory, learning motivation is defined as *the general goals students pursue during learning *(either mastery goals - gaining new knowledge; or performance goals - gaining a positive evaluation of competence or avoiding negative evaluation). Perceived psychosocial abilities are a desirable outcome, and low frustration tolerance (LFT), is a negative feature of student behavior. The hypothesis was that the mastery goal would be positively associated with psychosocial abilities while performance goals would be positively associated with LFT.

**Methods:**

143 first-year medical students completed at the end of an annual doctor-patient communication course a structured questionnaire that included measures of learning goal orientations (assessed by Pattern of Adaptive Learning Scale - PALS), psychosocial abilities (assessed by Psychological Medicine Inventory- student version -PMI-S) and Low Frustration Tolerance (LFT).

**Results:**

All study variables were found reliable (Cronbach's α ranged from .66 to .90) and normally distributed. Hierarchical multiple regression analysis revealed significant associations supporting the hypotheses. The mastery goal orientation was positively associated with perceived psychosocial abilities (PMI-S) (β = .16, p < .05) and negatively associated with low frustration tolerance (β = -.22, p < .05) while performance goal orientation was significantly associated with low frustration tolerance (β = .36, p < .001).

**Conclusions:**

The results suggest that the goal orientations theory may be a useful theoretical framework for understanding and facilitating learning motivation among medical students. Limitations and suggestions for practice within medical education context are discussed.

## Background

Professional conduct of physicians and medical students requires a high degree of psychosocial competence. Non-cognitive capabilities -- such as communication skills, psychological sensitivity, and the ability to successfully tolerate and cope with ongoing stresses and frustrations -- are essential for effective interactions with a wide range of patients and co-workers.

Medical curricula and residency programs in Western countries increasingly reflect the bio-psychosocial model proposed by Engel [1] and include significant behavioral science components. The curriculum that is currently advocated requires students to shift from the traditional bio-medical, disease-centered focus to a patient- or relationship-centered orientation [2]. However the integration of non-cognitive components in medical education may be challenging for many medical students. If students fail to comprehend the relevance of psychosocial topics to medical practice, their learning motivation will be poor, because motivation is often related to the relevance of learning processes [3]. In practice, students are often reluctant to study communication skills and may display initial (or even lasting) resistance and skepticism as they are instructed to change their orientations and behavior [4].

As psychosocial competence and frustration tolerance are important goals in healthcare professions, it is important to understand better how they might be enhanced in medical educational environments. Since all learning is driven by motivation (i.e. the willingness to exert effort toward educational goals, often despite difficulties and setbacks), we applied in the present study a motivational theory perspective: specifically, goal orientation theory. The aim was to investigate how self-reported psychosocial abilities and frustration tolerance might be associated with different motivational orientations among first-year medical students.

*Goal Orientation Theory *is a well-established perspective on students' motivation [5-7]. According to this theory, motivation can be defined by the general goal orientations students follow in the process of learning. For example, when two individuals engage in the same task; one may do so in order to demonstrate his/her abilities to others, while the other may wish to enjoy the activity and acquire new skills. These two individuals are expected to demonstrate different behaviors and affects toward the task. The first may invest effort only in tasks he/she will be able to perform well by demonstrating his/her relative abilities -- even by using unacceptable behaviors such as cheating [8] -- while the second may choose challenging tasks that will enable him or her to improve their knowledge and make significant inter-personal progress.

Scholars have attempted to classify and define those orientations. Within the goal-orientation literature, three goals are commonly known. In Mastery, the purpose is to acquire new knowledge or skills, characterized by those who enjoy merely participating in the act of learning. For example, a student who aspires to learn new concepts in Math or to solve equations that he did not succeed in the past, is considered as mastery orientated student. The goal of Performance-Approach is to gain a positive external evaluation, typified by those who wish to gain public recognition of their abilities. In this case the student will be eager to outperform his friend and to get recognition of relative abilities; his standard of success would be normative. In Performance-Avoidance, the purpose is to avoid negative external evaluation--those oriented as such wish to avoid being considered incapable. A student with this goal orientation will avoid situations in which his performance will be evaluated in relation to others [9,10]. These three goals are referred to as general orientations (i.e. general attitudes toward the task), rather than specific targets. Thus, when someone is mastery-oriented, his/her general approach toward the task would be general self-improvement, rather than attaining a specific target (e.g. improving course grades by 10 points). Conversely, when one is performance-approach orientated, he/she would focus on demonstrating superior relative abilities (e.g. getting the highest course grades). One with performance-avoidance orientation would make an effort to hide his/her lack of ability (e.g. avoiding participation or asking questions that may result in negative evaluation of his/her knowledge).

Recently, mastery-avoidance goal has also been discussed in the literature. It has been suggested that the purpose of this goal is to avoid deterioration of knowledge or skills that had been previously acquired. However, the definition and measurement of mastery-avoidance is still under dispute [11]; therefore, it was not included in the current study.

The findings regarding the benefits of goal orientations for various learning processes were consistent over the years and suggested that among the three orientations, the mastery orientation is the most adaptive performance goal in various learning environments [12]. For example, it was found that university students' levels of mastery goal orientation predicted their performance of a task requiring long-term retention of information. Conversely, performance-avoidance was negatively related to performance on that task [13]. Other studies found that students who reported holding mastery goals were more likely to use superior learning strategies such as deep information processing of new knowledge and elaboration (i.e. attempting to link the new information with old knowledge that had already been acquired) [14]. This was also found in relation to those who held performance goals and tended to use inferior strategies such as memorizing [15]. Such findings suggest that students who are orientated toward self-improvement will not just attempt to memorize the learning materials; rather they will strive to achieve better understanding of said materials, will make them more personally meaningful, and remember them for longer periods of time after learning.

It should be noted that the goal orientation theory was found applicable for many other contexts. Associations have been found between goal orientations and test anxiety [16], well-being in school (i.e. peer relationship, impulse control, and emotional tone) [17], inter-personal conflict solution strategies [18], establishment of intimate peer relationship [19] and even achievements [13,20,21]. These studies found that mastery goal orientations were more advantageous in the long term.

In the present study, we aimed to explore the usefulness of applying goal orientation theory to studying the effects of medical education, in the present case specifically, a physician-patient communication course. A recent study conducted in the context of medical education applied for the first time the goal orientations theory. Its findings supported the hypothesis that mastery goal orientations are likely to be associated with more beneficial outcomes in medical students' training, as compared to other goal orientations. In that study, mastery goal orientations were associated with positive outcomes (e.g. students' interest, effort, etc.) and were negatively associated with negative outcomes (e.g. tension and test anxiety) [22].

In the present study we explored possible associations between concepts from goal orientation theory and two potential outcomes of participation in a physician-patient communication course: desirable one - psychological medical abilities, and a negative one - low frustration tolerance.

*Psychosocial medical abilities *were defined as the level of interest, confidence, clinical abilities and sensitivity in addressing the psychosocial aspects of patient care [23]. This is an important aspect of students' interpersonal self-efficacy (i.e. the beliefs in one's future professional expertise as a physician) that was recently established as relevant to learning communication skills among medical students [24]

*Low frustration tolerance *(LFT) is a concept rooted in rational-emotive behavior therapy (REBT) models and denotes an irrational thinking pattern. REBT views psychological rationality as "that which aids and abets people achieving their basic goals and purposes"; and irrationality as "that which hinders or obstructs people from achieving their basic (long-range) goals and purposes" [25]. LFT specifically denotes intolerance of physical or emotional discomfort i.e. the inability to accept unpleasant physical sensations and symptoms, such as pressure, numbness and pain [26]. LFT is the tendency to exaggerate life's frustrations and inconveniences, viewing any form of discomfort as almost intolerable and to be avoided whenever possible. This tendency leads to dysfunctional behavioral consequences such as procrastination, resistance to change and lack of compliance with medical recommendations [26].

This conceptualization of LFT suggests that a high LFT score at the end of the communication course implies lower student ability to cope with demands and stress. Medical students as well as physicians are faced on a daily basis with countless stresses and frustrations. Successfully coping with these challenges is essential for effective professional functioning.

Based on the conceptualization of the mastery orientation as the more adaptive performance goal [10,12] and LFT as a dysfunctional discomfort avoidance tendency [26] we formulated the following hypotheses:

Hypothesis 1: The more adaptive goal orientation (mastery goal) would be found positively linked to desirable outcomes (in this case, perceived psychosocial ability) and negatively linked to the negative/maladaptive characteristic (low frustration tolerance, LFT).

Hypothesis 2: Conversely, the less adaptive goal orientations (i.e. performance-approach and performance-avoidance) would be found negatively linked to the desirable outcome (PMI) and positively linked to the maladaptive characteristic, LFT.

## Method

### Participants and procedure

143 first-year medical students from two classes in consecutive years responded to a structured questionnaire administered at the end of a mandatory physician-patient communication course. In this course the students learn and practice basic skills of medical interviewing, which focuses on two core components of medical interviewing: efficient information gathering and relationship construction [25]. The study was approved by the institutional ethics committee and participation was voluntary. Response rate was 96%.

Participants represented a relatively homogenous population of medical students in Israel, which were mostly mid- to high socio-economic status, with low variance of age, marital status and scholastic ability. The mean age was 22.2 (SD = 3.17) ranging between 17 and 30 years, 54.4% were female, and 94.1% were single.

### Measurements

*Goal orientations *were assessed using a self-report measure called Patterns of Adaptive Learning Scale (PALS), which previous studies found to be reliable and valid [10]. The assessment of mastery goal orientation included 5 items (sample item: "I like class work that I'll learn from even if I make a lot of mistakes"), performance-approach goal included 6 items (e.g. "I want to do better than other students in my class"), and performance-avoidance included 6 items (e.g. "It's very important to me that I don't look stupid in my class"). Responses were given on 5-point scales ranging from 1 "strongly disagree" to 5 "strongly agree". Because the measure was generic, the students were instructed to refer to that specific course. Internal reliability assessed by Cronbach's α in the current research was satisfactory, ranging from .62 to .89 (see table 1 for detailed descriptive statistics).

**Table 1:** Descriptive Statistics of study variables

Variable	N. items	Possible range	Mean (SD)	Skewness	Kurtosis	Cronbach's α
PMI	8	1-7	5.25 (.79)	-.31	-.15	.84
LFT	14	1-5	2.21 (.58)	.56	.31	.85
Mastery	5	1-5	3.93 (.49)	-.21	-.31	.66
P-Ap	6	1-5	2.46 (.85)	.10	-.88	.90
P-Av	6	1-5	1.91 (.61)	.30	-.14	.77

*Note*. PMI - Psychological Medical Inventory; LFT - Low Frustration Tolerance; P-Ap - Performance Approach; P-Av - Performance Avoidance.

*Perceived psychosocial abilities *were assessed using the Psychological Medical Inventory Student Version (PMI-S), which had been validated in previous studies [21]. The measure included 8 items with 7-point response scales ranging from 1 "not at all" to 7 "to a great extent". Sample items: "Awareness of how patients react to me"; "Confidence in dealing with psychological problems of patients". Internal reliability as assessed by Cronbach's α in the current research was high (.86) Low frustration tolerance (LFT) was assessed using a validated measure [22] that included 14 items with 5-point response scales ranging from 1 " strongly disagree " to 5 " strongly agree ". Sample items: "I find it difficult to tolerate discomfort or unpleasant conditions", "I get rather angry when someone keeps me waiting". Internal reliability in the current study was found to be .86.

### Statistical Analysis

Analysis was based on hierarchical multiple regressions, in which PMI and LFT were entered as dependent variables; the three goal orientations were entered as independent variables. The independent variables were entered in steps due to the high correlation between performance-approach and performance-avoidance goals. In that way it was possible to examine the separate contributions to the explained variance in the model. All variables were normally distributed as estimated by Skewness and Kurtosis. No significant associations were found between age or gender and any of the variables that were measured in this study. Therefore, age and gender were not included in the regression equations.

## Results

Descriptive statistics of study variables are presented in table 1. Students in general reported higher levels of psychosocial abilities (PMI) compared to frustration tolerance (LFT), the difference was significant in a paired-sampled t test (*t*(142) = 3 6.47, *p *<. 001), and higher levels of mastery goals compared to performance approach and avoidance (t(142) = 18.28, p < .001; t(142) = 30.03, p < .001, respectively). These results indicated that the students scored higher on adaptive and more desirable measures.

In order to reveal the pattern of relations between the study measures, we conducted a correlation matrix (table 2). As expected, scores on the mastery goal orientation measure were positively associated with scores on the PMI (r = .16, p < .05), and negatively related to the LFT level (r = -.21, p < .05). As expected, the performance goal orientations (P-approach and P-avoidance) were not related to the PMI, but were highly correlated with LFT.

**Table 2:** Correlation matrix of study variables

	PMI	LFT	Mastery	P-Ap
PMI	-			
LFT	0.04	-		
Mastery	.16 ^a^	-.21 ^a^	-	
P-Ap	.03	.40 ^c^	.05	-
P-Av	.01	.33 ^b^	-.07	.66 ^c^

*Note*. PMI - Psychological Medical Inventory; LFT - Low Frustration Tolerance; P-Ap - Performance Approach; P-Av - Performance Avoidance. (^a ^*p *<.05, ^b ^*p *<.01, ^c ^*p *<.001; two-tailed).

In order to examine whether these relations remained stable after control of the other variables, they were entered into a linear regression (table 3). It is important to note the high correlation found between P-Ap and P-Av (*r *= .66, p < .001). This association may indicate that the students in the present sample did not differentiate between the two goals and perceived them as one. The small range of discrepancies between the two performance goals and all the other variables (from .02 to .07) supports that premise. Therefore, a hierarchical linear regression analysis was implemented in which the first step included Mastery and P-Ap as predictors, and P-Av was entered in the second step. This procedure enables examination of the changes in the relations of the goal orientations to the outcome.

**Table 3:** Predictors of PMI and LFT *- *results of a hierarchical linear regression

	PMI Step 1	Step 2	LFT Step 1	Step 2
Mastery	.16 ^a^	.16 ^a^	-.23 ^b^	-.22 ^b^
P-Ap	.02	.02	.41 ^c^	.36 ^b^
P-Av		.01		.09

R^2^	.03	.03	.21 ^c^	.22 ^c^

*Note*. ^a ^*p *<.05, ^b ^*p *<.01, ^c ^*p *<.001

Results from the regression analysis supported the hypothesis that the Mastery goal orientation was positively associated with PMI, and P-Ap was associated with LFT, even after controlling for the other goal orientations. Entering P-Av to the equation did not change the result, indicating that P-Ap is a more significant predictor of LFT. The results of the multiple regression analysis (table 3) provide further support for the association between achievement goals and student perceptions: The mastery goal orientation was significantly associated with perceived psychosocial abilities (PMI-S) (β = .16, p < .05) but negatively associated with LFT (β = -.23, p < .01); and performance goal orientation was significantly associated with low frustration tolerance (β = .36, p < .01).

## Discussion and Conclusions

The main purpose of the current study was to explore whether goal orientation theory can provide a constructive framework for understanding medical students' perceived psychosocial attributes. Previous studies have shown that goal orientations can predict positive and negative outcomes in medical education such as interest and test anxiety [16]. In order to provide additional empirical evidence to support the benefits of applying goal orientation theory perspective to research in medical education, we tested the associations between students' goal orientations and two possible outcomes in their communication skills training: PMI and LFT. We hypothesized that the adaptive pattern of motivational orientation (mastery goal) would be positively associated with the more adaptive outcome (PMI), while the less adaptive goal orientation (performance goals) would be associated with the maladaptive outcome (LFT).

The findings supported the hypothesis, as the mastery goal orientation was highly positively correlated with PMI. Therefore, students who pursue interpersonal goals in learning, striving to acquire knowledge and improve their skills in that domain, are more likely to report better perception of their ability to cope with their patients' psychosocial needs.

On the other hand, as expected, the less adaptive pattern of goal orientations was correlated with the maladaptive outcome in this course: performance-approach goal orientation was highly positively correlated with LFT. That means that students with more interpersonal goal orientations in learning, those who wish to attain positive external evaluation of their abilities, are more likely to report lower perceived ability to deal with stress and tolerate frustrations.

Such findings suggest that applying a goal orientation theory perspective to medical education research may be beneficial to our understanding of psychosocial attributes among medical students. Furthermore, this literature can be used to draw practical implications for teaching in medical contexts. For example, it is possible to design specific interventions to elicit more mastery goal orientations among medical students [6,20]. As our findings suggest, it is important to reinforce mastery goals, since this orientation is associated with desirable outcomes (for instance PMI in the current study).

Although the current course did not apply goal orientation theory, we will elaborate about possible application for prospective practice and research in medical training. One of the most prominent models that explain student choice in goals is called TARGET (task, authority, recognition, grouping, evaluation and time) [6,27]. *Tasks *should be designed in a manner that will be relevant to the students and will allow them to express their abilities in various ways (i.e. not only multiple choice written exams, but also alternative measures to demonstrate competence). *Authority *should be constructed in order that staff and students would communally share the responsibility for self-improvement (symmetric accountability). *Recognition *of students' performance should be based not solely on performance results, but also on effort and commitment to the learning process. *Grouping *students for collaborative assignment can reduce competitiveness and enhance motivation. *Evaluation *should not overtly rank the student, but rather remain confidential. *Time *should be dedicated mostly for learning and less for assessments. In her studies, Ames [6,27] investigated the model by using various design studies (correlational and experimental) in order to substantiate the causal relations.

Of note, in the present study the students reported higher levels of mastery goals than performance goals. This finding suggests that the medical students in the present sample reported higher levels of the positive measures in comparison to the negative measures. This trend should be tested in prospective studies to determine whether it persists, since goal orientations tend to be subject to change over time [28]. Further studies with larger samples of medical students should investigate whether this trend is replicated.

It is important to note that these recommendations are subject to the limitations of the current study. First, future studies should aim to establish causal relationships; and then investigate whether goal orientations induce the hypothesized outcomes, and not vice versa. In addition, the models tested in the study include merely the goal orientations; as such the total shared variance of the model ranged from 3 to 21%. Even though the results were found to be significant, future studies should include other factors that may provide more substantial models (e.g. personal traits such as fear of failure, or environmental characteristics such as teacher facilitation of goals).

Despite these limitations, the results of our study suggest that the use of goal orientation theory in studies of medical education offers an additional perspective that may lead to a better understanding of the factors that enhance the effectiveness of medical student training.

## Competing interests

The authors declare that they have no competing interests.

## Authors' contributions

NM substantially contributed to conception and research design, analysis and interpretation of data; drafting and revising the article. YGB substantially contributed to conception and research design, acquisition of data, and analysis and interpretation of data; drafting the article and then revising it critically for important intellectual content. TK substantially contributed to conception and research design, acquisition of data, and analysis and interpretation of data; drafting the article and then revising it critically for important intellectual content. All authors read and approved the final manuscript.

## Pre-publication history

The pre-publication history for this paper can be accessed here:

http://www.biomedcentral.com/1472-6920/12/4/prepub
